# Recent Advances in the Development of Antimicrobial and Antifouling Biocompatible Materials for Dental Applications

**DOI:** 10.3390/ma14123167

**Published:** 2021-06-09

**Authors:** Poornima Ramburrun, Nadine A. Pringle, Admire Dube, Razia Z. Adam, Sarah D’Souza, Marique Aucamp

**Affiliations:** 1 School of Pharmacy, Faculty of Natural Sciences, University of the Western Cape, Cape Town 7535, South Africa; 4078543@myuwc.ac.za (N.A.P.); adube@uwc.ac.za (A.D.); maucamp@uwc.ac.za (M.A.); 2 Department of Restorative Dentistry, Faculty of Dentistry, University of the Western Cape, Cape Town 7505, South Africa; rzadam@uwc.ac.za

**Keywords:** antimicrobial and antifouling materials, dental restorative materials, polymers, biofilms, periodontitis, recurrent caries

## Abstract

The risk of secondary bacterial infections resulting from dental procedures has driven the design of antimicrobial and antifouling dental materials to curb pathogenic microbial growth, biofilm formation and subsequent oral and dental diseases. Studies have investigated approaches based primarily on contact-killing or release-killing materials. These materials are designed for addition into dental resins, adhesives and fillings or as immobilized coatings on tooth surfaces, titanium implants and dental prosthetics. This review discusses the recent developments in the different classes of biomaterials for antimicrobial and antifouling dental applications: polymeric drug-releasing materials, polymeric and metallic nanoparticles, polymeric biocides and antimicrobial peptides. With modifications to improve cytotoxicity and mechanical properties, contact-killing and anti-adhesion materials show potential for incorporation into dental materials for long-term clinical use as opposed to short-lived antimicrobial release-based coatings. However, extended durations of biocompatibility testing, and adjustment of essential biomaterial features to enhance material longevity in the oral cavity require further investigations to confirm suitability and safety of these materials in the clinical setting. The continuous exposure of dental restorative and regenerative materials to pathogenic microbes necessitates the implementation of antimicrobial and antifouling materials to either replace antibiotics or improve its rational use, especially in the day and age of the ever-increasing problem of antimicrobial resistance.

## 1. Introduction

According to the World Health Organization (WHO), oral diseases affect nearly 3.5 billion people worldwide [1]. The hard and soft tissue surfaces of the oral cavity provide the ideal environment for abundant microbial growth and biofilm formation (also known as dental plaque) [2]. With increased attraction of dental reconstructive and replacement procedures—microbial growth and biofilm formation has extended its territory to the surfaces of the artificial materials implemented in these restorative applications. The use of long-wear dental materials intensifies the clinical management of pathogenic microbes and increases the risk for oral disease such as dental caries, periodontitis, peri-implantitis, mucosal disease and oral cancer [3]. Tooth-loss, disfigurement, pain and difficulties in eating and speaking associated with inefficient oral hygiene ensuing oral disease cause significant psychological and social distress in afflicted patients [4].

Amid concerns over microbial resistance, there is a plea to develop novel antimicrobial compounds as well as to reduce unnecessary antibiotic dental prescriptions to prevent the emergence of resistant pathogens [5,6]. Antiseptic cavity cleaning solutions and oral rinses paired with aerated-water sprays and mechanical cleaning (professional and daily teeth-brushing with fluoridated products) are commonly used for chemical bacterial elimination and physical detachment of bacteria from the tooth and dental material surface, respectively [4,7]. Lack of regular oral hygiene, inadequately cleaned dental prosthesis and the continuous accumulation of bacteria makes it impossible to completely eliminate the microbial burden in the oral cavity.

It has been realized that restorative dental materials capable of bacterial growth inhibition and prevention of microleakage (between the tooth-material interface) is needed to prevent recurring infections, recurrent caries and failure of dental restorative procedures [8,9]. Such antimicrobial and antifouling materials either release components which kill microbes upon contact or interrupt and prevent microbial attachment and biofilm formation, respectively. This led to the development of the first commercial antimicrobial dental bonding resin system in 1994—the bactericidal 12-methacryloyloxydodecylpyridinium bromide (MDPB) monomer currently used in the product CLEARFIL SE Protect [10,11]. Since then, MDPB-immobilized resinous materials have been common in dental reconstructive and restorative procedures to curb the occurrence of secondary bacterial infections and subsequent oral diseases [12].

Antimicrobial and antifouling dental materials offer a promising role in the prevention of secondary caries, demineralization processes and implant failure occurring from biofilm formation on dental tissues and the dental materials utilized [12]. Transition to these materials may further ascribe to simpler daily maintenance of oral hygiene and longer wear resistance of dental materials. The advancement of antimicrobial and antifouling dental materials includes a variety of release-based and immobilization strategies comprising: (1) antibiotic and antiseptic compounds; (2) metal ions; (3) polymeric biocides and (4) antimicrobial peptides from a host of dental resins, adhesive systems, implant coatings and biodegradable polymeric drug delivery devices.

The challenge in developing antimicrobial and antifouling dental materials for clinical use lie in the pursuit of achieving the optimal features of biocompatibility, bioregeneration and rechargeability—without compromise on mechanical properties, surface properties and aesthetic appeal [13,14]. Additional essential features of antimicrobial and antifouling materials for dental applications are elaborated in Table 1.

This paper provides an overview of the emerging trends and developments in antimicrobial and antifouling biomaterials for various dental restorative, reconstructive and replacement strategies. Concerns persist surrounding the toxicity of leachable unreacted monomers from partially cured resin systems, despite their established antimicrobial efficiency. As such, cytocompatibility appeal is highlighted, where possible, in these developments as it is a pertinent property to approve any material for implantation or use in the human body, particularly the oral cavity. The types of microbial susceptibility and the challenges associated with restorative, prosthetic, endodontic and regenerative materials are also expressed.

## 2. Microbial Susceptibility in Dental Materials 

Dental restorations and implants that are introduced into the oral cavity have the potential to alter the physiological, chemical and mechanical environment of the oral cavity, thereby creating new niches for various microorganisms [29]. Dental materials are thus vulnerable to aggressive bacterial attack once placed in the oral cavity [30]. The microbial susceptibility of dentures and other dental materials is primarily influenced by the topography and surface chemistry of the material. Dental caries or cavities are identified as the dominant chronic infectious disease within the oral cavity where the rate of dental cavity formation is estimated to be as high as 50–60% following restorative treatment. The primary cariogenic bacteria responsible for cavity formation include *Streptococcus mutans*, *Lactobacilli* spp., *Actinomyces* spp. and other anaerobic bacteria [31,32]. The adherence of such cariogenic bacteria to dental materials results in the biodegradation of these materials. Furthermore, cariogenic bacteria promote the development of secondary caries—one of the principal causes of glass-ionomer cement and composite resin restoration degradation and failure—as well as peri-implantitis [30,32]. Respiratory pathogens, such as *Streptococcus pneumoniae*, have also been shown to colonise dentures of the elderly population indicating that denture plaque may act as a reservoir for potential respiratory pathogens [33].

Likewise, dental materials are also prone to fungal contamination as fungi are effectively able to adhere to various resin, glass and metal surfaces. Fungal growth has been shown to degrade denture liners and subsequently cause tissue irritation. Individuals wearing dentures thus appear to be particularly susceptible to fungal infection as there is a strong correlation between the presence of *Candida albicans* and denture stomatitis [34,35]. The susceptibility of dental materials to viral infections appears less well studied, however, several viral species have been found in the oral cavity with human herpes viruses being the most common. Viruses are suspected to be involved in the development of various ulcers, tumours and infectious diseases in the oral cavity as well as periodontitis [36].

**Figure 1:**
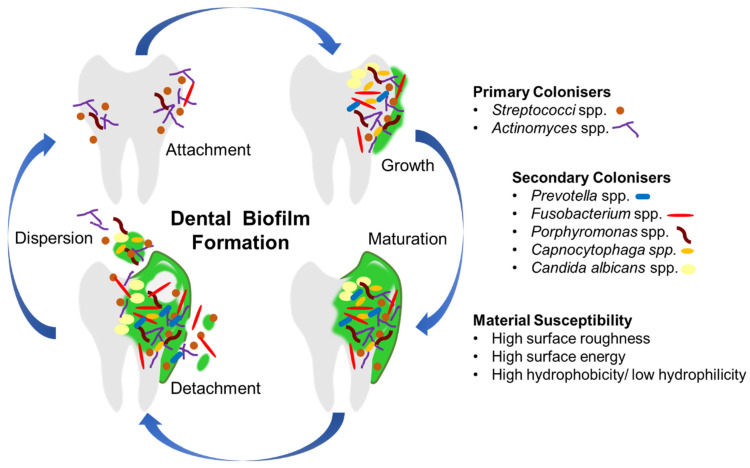
Schematic representation of primary dental microbes and biofilm formation.

Bacteria, fungi and viruses seldom grow separately from one another in the oral cavity and instead tend to form consortiums that result in the formation of biofilms. A biofilm, such as dental plaque, is an aggregation of various microorganisms organised in thin layers and embedded in a self-produced matrix consisting of extracellular polymeric substances (EPS) which can form on natural dental tissues as well as the surface of various dental materials [29]. The formation of biofilms occurs through a multi-step process involving microbial attachment followed by biofilm maturation and biofilm dispersal, as depicted in Figure 1 [37]. Every surface within the oral cavity is covered by a pellicle of glycoproteins (naturally present in saliva) and it is to this layer of glycoproteins that microorganisms can adhere. During biofilm formation, Gram-positive bacterial species such as *Streptococcus* spp. and *Actinomyces* spp. are referred to as pioneer species as they are generally the first microorganisms to adhere to the dental pellicle. These species thus perform a crucial role in creating and sustaining conditions that enable other microorganisms to colonise and thrive on dental surfaces and materials. Their respiration process creates hypoxic conditions that make it conducive for the growth of various anaerobic species [38]. 

The role of oral biofilms in pathogenesis is dependent on the composition and the location of the biofilm. Biofilms containing microorganisms that produce acid at the tooth-restoration margin may cause recurrent caries as well as pulp infections; biofilms persisting in the root canal system following root canal therapy may cause re-infection as well as persistent apical periodontitis. Biofilms growing on periodontal tissues and dental implants could result in periodontitis and peri-implantitis whereas biofilms on dentures are associated with malodour, aspiration pneumonia and various systemic conditions—particularly amongst the elderly and dependent [34,37]. Oral biofilms are difficult to remove as these communities of microorganisms act as a single unit within the EPS matrix which protects them from chemical treatments. Limited access to surfaces between the teeth and deep crevices on the tooth makes it difficult to expel these microbial colonies. This situation further complicates the clinical applications of dental materials due to the presence of pores, margins and fissures on both the material and tissue surfaces [29].

## 3. The Role of Antimicrobial and Antifouling Materials in Clinical Dental Applications

### 3.1. Dental Luting Cements

**Figure 2:**
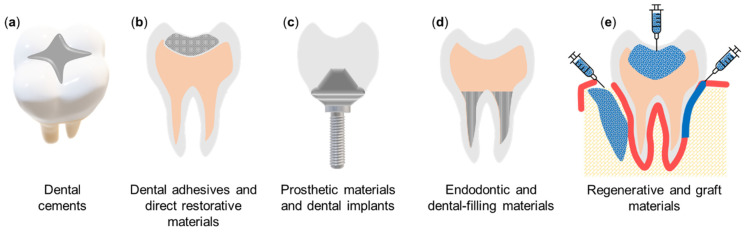
Illustrations of dental material applications that require antimicrobial and antifouling properties: (**a**) dental cements; (**b**) dental adhesives and direct restorative materials; (**c**) prosthetic materials and dental implants; (**d**) endodontic and dental-filling materials; (**e**) regenerative and graft materials.

Dental luting cements are used to connect indirect restorations permanently in the mouth (Figure 2a) by filling the void between the restoration-tooth interface to avoid dislodgement during mastication [39]. Failure of crowns and fixed partial dentures may be attributed to a loss of crown retention. Recurrent caries is also commonly implicated in the failure of fixed partial dentures. Dissolution of dental cements at the margins may result in marginal microleakage and the accumulation of cariogenic bacteria. Similarly, in orthodontics, the accumulation of cariogenic bacteria around the margins of fixed orthodontic apparatus results in enamel demineralisation and the appearance of white spot lesions which occur in 50% of patients [40]. 

In addition to high compressive strength and stiffness, luting cements should be translucent, biocompatible and anticariogenic [41]. The introduction of antimicrobial and antifouling dental cements may reduce the failure of fixed restorations by eliminating the bacterial colonies that remain underneath the restoration and preventing microleakage between the cement-tissue interface [42]. To confer antimicrobial activity, most studies investigate the incorporation of metallic nanoparticles of silver, zinc oxide or titanium dioxide into glass ionomer luting cements [42,43,44] or copper-containing glass ionomer and zinc oxide phosphate cements [45,46]. Other methods have explored the inclusion of chlorhexidine and propolis [47] as well as contact killing cationic quaternary ammonium compounds (QACs) in glass ionomer cements [48].

### 3.2. Dental Adhesives and Direct Restorative Materials

Dental composite resin materials (Figure 2b) are commonly used to restore teeth. However, these tend to accumulate more biofilm compared to other dental materials and experience faster degradation due to microbial growth and biofilm formation as result of being in direct contact with oral mucosal surfaces and salivary proteins [40,49]. Both adhesive and composite resins are also susceptible to polymerisation shrinkage upon light-curing which leads to the development of gaps at the tooth-restorative interface, thus creating a pathway for bacterial ingress, colonisation and induction of secondary caries. Recurrent caries is the most common reason for the replacement of dental restorations [50]. The replacement or repair of restorations may lead to compromising of remaining tooth structure and could eventually result in the loss of a tooth [51].

The prevention of plaque accumulation on tooth and restorative surfaces is clinically attempted with (1) pre-treatment disinfectants and oral cavity cleansers (2) the incorporation of leachable antibacterial agents and (3) the inclusion of polymerised or filler particles of QACs into the bonding agents. Pre-treatment of teeth and leachable agents commonly comprise chlorhexidine, glutaraldehyde and benzalkonium chloride as disinfectants in the formulations [11,52,53].

Most of the available commercial dental resin composites are not antibacterial as they are composed of inert inorganic fillers and organic monomers. Some authors have suggested that dental composites may encourage bacterial growth due to the release of ethylene glycol dimethacrylate and triethylene glycol dimethacrylate [54]. Although limited, there are commercially available adhesive systems that have shown antibacterial activity for clinical use in dental applications, such as Clearfil SE Protect (containing MDPB) and GLUMA 2Bond (containing 5% glutaraldehyde).

Clearfil SE Protect demonstrated good bactericidal activity against several facultative and strict anaerobic microorganisms without reducing dentine bond strength while controlling caries progression in enamel [11,53]. Studies have indicated that MDPB displayed moderate cytotoxicity in mouse fibroblasts, odontoblast-type cells and human pulpal cells and is considered acceptable for use in dental applications [55,56,57]. However, concerns have been raised around the biocompatibility of GLUMA 2Bond due to the cytotoxicity of the aldehyde moiety [53]. Both products eradicated the common cariogenic and periodontic pathogens *S. mutans*, *Fusobacterium nucleatum*, *Porphyromonas gingivalis*, *Prevotella intermedia*, *Prevotella nigrescens*, *Enterococcus faecalis* and *Lactobacillus casei* [53,58].

The addition of filler particles such as silver, zinc oxide, copper iodide and bioactive glass as well as the polymer chitosan to experimental dental adhesive systems have been reported to impart antibacterial properties [52]. However, a drawback to the use of metallic ions is the resulting colour, which diverges from the natural tooth colour and negatively affects its aesthetic appeal [52,59].

### 3.3. Prosthetic Materials and Dental Implants

The popularity of dental implants has been escalating over the years as it is the preferred treatment option for the replacement of single or multiple missing teeth (Figure 2c) [60]. Despite its success, aseptic loosening and infection of implants are the common reasons for clinical failure [61]. The intraoral component of a dental implant is surrounded by gingival crevicular fluid, food debris, saliva, sugars and bacterial metabolites. This creates a microenvironment conducive for microbial growth, particularly for Gram-negative pathogens, which may cause peri-implantitis and periodontitis. Antifouling polymeric coatings deposited onto the surface of metallic screw-in components may enhance the longevity of dental implants by preventing microbial colonisation, infection and subsequent implant failure.

The use of removable partial and complete dentures acts as reservoirs of microorganisms in the mouth. The colonisation of the surfaces of these prostheses put them in constant contact with the oral mucosa. Most commonly, denture stomatitis results from poor oral hygiene and poor denture habits [62]. It is estimated that approximately 70% of denture wearers suffer from denture stomatitis [63]. The use of an antimicrobial denture base material and antifouling coatings may prevent future recurring infections by managing microbial colonisation and inhibiting biofilm formation, respectively, on the surfaces of these prosthetics. In addition, it would assist denture-wearers in maintaining good oral hygiene and health by preventing the onset of dental diseases. Denture base materials may be transformed into an antimicrobial substrate with the addition of silver and titanium dioxide nanoparticles, chlorhexidine, or the immobilisation of QACs [64]. However, the undesirable leaching of these compounds may adversely affect the health and viability of soft oral tissues and so material properties of water solubility and water sorption should be carefully modulated.

### 3.4. Endodontic and Dental-Filling Materials

The purpose of endodontic treatment is to remove the source of infection in the root canal space (Figure 2d). The root canal treatment process involves instrumentation, irrigation and intracanal medication. This process aims to remove all microorganisms and their products. However, studies have reported that bacteria may remain in the dentinal tubules and cementum which may become sources of inflammation and infection [65]. *E. faecalis* is the most prevalent microorganism in infected root canals and has the ability to invade deeply into dentinal tubules while resisting intracanal procedures and surviving in filled canals [66]. The development of endodontic medicaments, intracanal posts and resins for core build-ups with antibacterial properties would assist in the repair of apical and periapical tissues [67].

A variety of commercial root canal sealers are available. Antimicrobial agents found in current endodontic sealers include eugenol, zinc oxide, thymol, hexamethylenetetramine, calcium oxide and paraformaldehyde [68]. These components may exhibit toxic effects to host tissues as they lack selective toxicity to microorganisms [66]. Kapralos et al. investigated the antibacterial activity of four commercially available endodontic sealers against Gram-positive planktonic and biofilm bacteria: AH Plus, TotalFill BC Sealer, RoekoSeal and Guttaflow 2 [69]. RoekoSeal displayed no antimicrobial effect. Whilst Guttaflow 2, a polydimethylsiloxane-based sealer, displayed no antibacterial effect despite containing silver microparticles. The commercial sealers AH Plus and TotalFill BC Sealer showed adequate antibacterial properties. The epoxy resin-based sealer, AH Plus, may impart bactericidal activity resulting from the release of formaldehyde constituents during setting [69]. Similarly, methacrylate-resin based sealers may exhibit antibacterial properties due to the low pH and elution of non-reacted monomers [70]. Silicate-based sealers produce calcium silicate hydrogels and calcium hydroxide during the hydration reaction thereby conferring antibacterial properties to the sealer [66].

### 3.5. Regenerative Materials

The primary motive of using dental regenerative materials is in periodontitis for the formation of new bone and supporting connective tissue (Figure 2e) [71]. Periodontitis is an inflammatory disease affecting the supporting structures of the teeth such as the periodontal ligament, bone and gingiva [72]. If left untreated, periodontitis could eventually lead to permanent tooth loss and systemic inflammation [73]. Treatment for periodontal disease includes debridement, radicular conditioning, bone grafting/substitution and guided tissue regeneration in conjunction with the oral administration of antibiotics [74]. As a result of the disease or during treatment processes, root surfaces may be exposed to many bacteria. The success of tissue regeneration may be compromised by local infections caused by microorganisms present in the wound area; therefore, treatment strategies include antibiotic therapy to reduce the risk of wound contamination. Currently, the development of new multifunctional materials to encourage tissue regeneration and minimise local microbial infections are required to improve patient treatment outcomes [71]. Most biomaterials intended for the treatment of periodontitis constitute polymeric advanced drug delivery systems (comprising polymeric nanoparticles, nanofibers, films, chips and thermo-responsive gels) [75] for the sustained release of conventional antibiotic drugs (tetracyclines, metronidazole, clindamycin, ciprofloxacin) [74] into the periodontal intrapocket as targeted therapy. The use of polymeric materials combined with growth factors to promote and support tissue regeneration and healing, may further complicate treatment strategies and lead to treatment failure as they are highly susceptible to microbial accumulation and biofilm formation [76]. The development of novel antimicrobial and antifouling materials, or their inclusion into polymeric composites under investigation, may further assist in reducing the high antibiotic loading dose required in drug delivery applications. This would thereby allay the progression of microbial or antibiotic resistance whilst maintaining a low-microbial environment supportive of healthy tissue regrowth.

## 4. Drug-Releasing Antimicrobial Dental Coatings and Resins

Drug-releasing dental biomaterials constitute a range of polymeric carriers in the form of gels, films, scaffold implants, electrospun nanofibers, nanoparticles or combinations thereof for the controlled release of therapeutic compounds. Common therapeutic compounds for dental applications include antibiotics such as metronidazole, ciprofloxacin, tetracyclines and chlorhexidine as well as non-steroidal anti-inflammatory agents [77]. Most drug-releasing dental biomaterials are designed for the infectious and regenerative treatment of periodontitis whereas others are applied in the synthesis of antibacterial coating materials, dental composites, sealers and adhesives [75,78,79,80,81]. Although appropriate for the treatment of periodontitis, drug-releasing dental biomaterials for restorative applications face the challenge of long-term stability, duration of antimicrobial activity and systemic drug exposure. The duration of antimicrobial activity depends on the quantity of drug available for release within the material. Drug elution could also compromise the mechanical integrity of the materials via formation of voids and weakened porous networks after exhaustion of drug release. Coatings are preferred as they do not compromise the mechanical properties of materials and may be reapplied to certain surfaces, such as tooth enamel and exposed implants, when required [82].

### 4.1. Chlorhexidine-Releasing Systems

Over several years, chlorhexidine has remained the gold standard in combating bacterial infections of the oral cavity and is commonly used in mouthwashes at low concentrations as well as in dental composites for antibacterial effect [78]. Concerns of cytotoxicity leading to tissue inflammation, tissue necrosis and reduced regenerative capacity of gingival fibroblasts and periodontal tissues has motivated investigations into long-term chlorhexidine-releasing materials [83,84]. Prolonged and localised delivery of chlorhexidine from dental materials embedded within systems such as titanium coatings, tooth enamel coatings, dental resins and composites using biocompatible polymers may reduce cytotoxicity of the drug whilst maintaining adequate antimicrobial and antibiofilm activity. Wood et al. reported sustained drug release from chlorhexidine hexametaphosphate nanoparticles coatings over 99 days due to retention of the nanoparticles within the salivary pellicle coated onto titanium surfaces whereas another group reported drug release over 7 days from coated surfaces of hydroxyapatite discs [85,86]. Similarly, chlorhexidine has been loaded into porous silica nanoparticles within biocompatible polymeric coatings of polydimethylsiloxane and pH-sensitive polyvinyl pyridine [82,87]. It was reported that an extended 1-week washing period of the nanoparticles could reduce cytotoxicity against gingival fibroblasts by removing surface adsorbed chlorhexidine molecules [87].

Chlorhexidine incorporated into dental resins typically exhibits sustained drug release over a range of 25–29 days [88,89,90,91,92]. Chlorhexidine-loaded montmorillonite composites were shown to minimise interference with mechanical properties (elastic modulus, flexural strength and degree of conversion) of (Bisphenol A bis(2-hydroxy-3-methacryloxypropyl) ether and triethyleneglycol dimethacrylate resin matrices [92]. One group of researchers focussed on the design of antibacterial resin-dentin bonding systems for chlorhexidine delivery inside dentinal tubules of demineralized dentin via polycaprolactone nanocapsules and poly lactic-co-glycolic acid nanoparticles as drug carriers [88,89]. Cytocompatibility testing over 72 h showed maintenance of dental pulp stem cell viability at >80% whereas reduced viability of <60% was observed for the maximum level of chlorhexidine-loading in a dose-dependent manner [89].

Whereas the aforementioned delivery systems rely on polymer erosion and diffusion for drug release—Luo and co-workers designed ultrasonic and magnetic controlled chlorhexidine drug delivery systems within urethane dimethacrylate-hydroxyethyl methacrylate (UDMA-HEMA) resins [90,91]. Chlorhexidine-loaded microspheres embedded into UDMA-HEMA resins provided ultrasonic-triggered biphasic drug release over 27 days: constituting a stage of slow release over the first 8.5 days followed by a rapid release over the remaining 18.5 days [91]. The magnetic controlled release system was synthesised by integrating magnetic Fe_3_O_4_ nanoparticles into chlorhexidine crystals. The Fe_3_O_4_-chlorhexidine 0.0005% UDMA-HEMA resins were able to maintain fibroblast cell viability at >80% whilst bacterial viability was practically eliminated [90]. The Fe_3_O_4_ component alone was shown to be non-cytotoxic with >100% fibroblast viability and hence it reduced chlorhexidine-induced cytotoxicity. This system is considered to act via a dual antimicrobial mechanism involving (1) the chemical killing action of chlorhexidine and (2) the physical killing action of the metallic nanoparticles. Sustained drug release was achieved over 27 days with cumulative release rates in the range of 4.4–7.4% where drug release increased upon exposure to a magnetic field. This magnetic-guided drug delivery approach is suggested for incorporation into dental resins, filling materials and denture linings whereas its incorporation into gels and varnishes could be applied for targeted treatment of periodontitis and peri-implantitis [90].

### 4.2. Antibiotic-Releasing Systems

**Figure 3:**
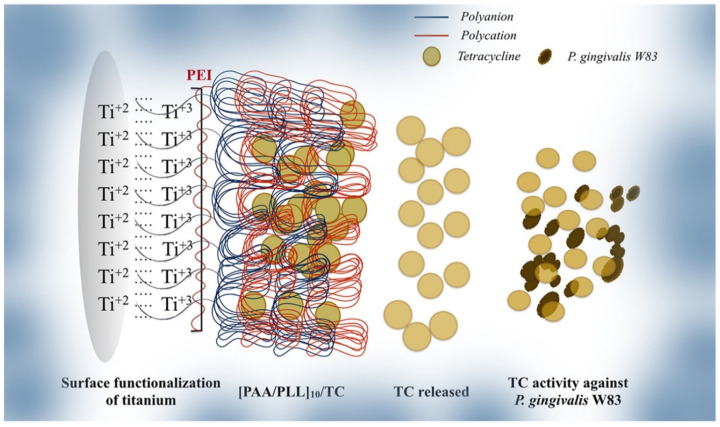
Illustration of the surface functionalization of layer-by-layer polyelectrolyte depositions of PAA and PLL on titanium implants for the sustained release of tetracycline [95]; reproduced with permission from [95], Elsevier B.V. Ltd., 2019.

Due to their architectural resemblance to extra-cellular matrices of dental tissues, electrospun nanofibers loaded with antibiotics such as tetracycline, gentamicin and metronidazole have shown to favour cellular regeneration whilst offering antibacterial activity via release of drug molecules [77,93,94]. Such systems are investigated primarily as antibacterial coatings for titanium implants for the prevention of peri-implantitis. The advantages of promoting cellular interactions of adhesion and proliferation at the titanium-coating interface assists with implant integration within the host tissues. Tetracyline-loaded nanofibers at 25% *w*/*w* blend of poly(DL-lactide), poly(ε-caprolactone) and gelatine were shown to inhibit biofilm formation and enhance cell proliferation attributed to the activity of tetracycline [77,93]. Likewise, non-fibrous thin film coatings of polylactic acid-hydroxyapatite-gentamicin and layer-by-layer polyelectrolyte deposition of poly(acrylic acid) (PAA) and poly-l-lysine (PLL) coatings on titanium surfaces (Figure 3) also supported cellular proliferation, proving non-cytotoxicity and antibacterial activity [79,95].

## 5. Antimicrobial Nanoparticle-Based Strategies

**Figure 4:**
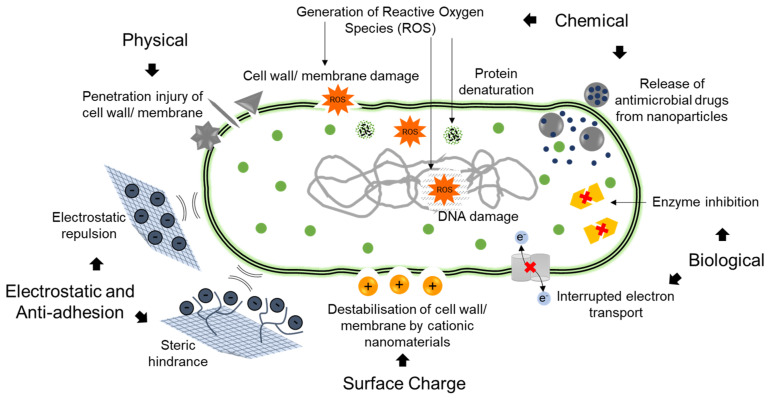
Schematic representation of the antimicrobial and antifouling mechanisms of nanomaterials: physical, chemical, biological, surface charge properties, electrostatic and anti-adhesion properties.

Nanoparticles (particles within a 1–100 nm size range) have been proposed for the treatment of chronic bacterial infections as drug carriers in both a local and systemic drug delivery context [96,97]. The antimicrobial agent may either be encapsulated within or conjugated onto the surface of the nanoparticle. Drug cargo can be released from the nanoparticles in a sustained or controlled fashion providing the potential for constant drug levels above the minimum inhibitory concentration (MIC) for sustained killing of the microbes. The material composition of the nanoparticle may also inherently possess antimicrobial and antifouling activity. Such antimicrobial and antifouling mechanisms of nanoparticles are summarised in Figure 4. Recent advances in the composition and design of the nanoparticles have created ‘smart’ nanoparticles, which are stimuli responsive, capable of mimicking the bacteria in form and function (i.e., biomimetic nanoparticles), incorporating theranostic functionality and modulating immune responses in the host [98,99].

Materials selected to synthesize polymeric nanoparticles may possess bioadhesive properties enabling prolonged adhesion of the nanoparticles to the tissue in the periodontal pocket (for the treatment of periodontitis) or mucosa. Examples of bioadhesive polymers include chitosan, dextran and polyacrylic acid [100]. Nanoparticles can be delivered directly into the periodontal pocket or loaded into films which can be inserted into the periodontal pocket [101]. In relation to the treatment of periodontitis, polymeric nanoparticles and metallic nanoparticles are among the most explored materials to achieve drug delivery and immuno-modulation, as well as antimicrobial action. Polymeric nanoparticles have been under intense investigation in recent times given their greater biocompatibility in relation to metallic nanoparticles [102,103].

### 5.1. Metallic Nanoparticles and Antimicrobial Activity

Metallic nanoparticles, for example silver (Ag) and the metal oxides of nanoparticles of iron, zinc and titanium are known to disrupt pathogen membranes and generate reactive oxygen species (ROS) which cause mitochondrial damage, cell membrane damage and protein denaturation (as shown in Figure 4) [104]. Other mechanisms of action include inhibition of metabolic processes, displacement of magnesium ions required for the enzymatic activity of oral biofilms, disturbance of the electron transportation, oxidation of macromolecules and prevention of DNA replication [105,106,107,108]. The development of resistance is associated with the ability of pathogens to form biofilms, and metallic nanoparticles have widely been investigated as agents to penetrate the biofilm and achieve bacterial reduction.

To reduce cytotoxicity of metallic nanoparticles, green synthesis methods have been employed [109,110,111]. Several studies have successfully synthesized silver and copper nanoparticles from fungi which are able to reduce the starting materials to produce nanoparticles with antibacterial and anti-inflammatory properties [112,113,114]. Silver nanoparticles synthesized from the fungi *Fusarium semitectum* were found to be effective against *P. gingivalis*, *Bacillus pumilus*, and *E. faecalis* in vitro [115]. This activity was comparable to that of chlorhexidine. These nanoparticles were also found to be compatible against a human gingival fibroblast cell line [116]. Microbial methods for the synthesis of metallic nanoparticles may be useful for the development of non-toxic, antimicrobial and bioactive dental nanomaterials and this should be explored further in dental pathogens.

To overcome the challenges of chlorhexidine inactivation and cytotoxicity toward human cells, Tokajuk et al. synthesized aminosilane-coated magnetic iron oxide nanoparticles functionalized with chlorhexidine [117]. In the presence of human saliva, the nanoparticles displayed significantly greater bactericidal activity against biofilm-forming microorganisms, including *Pseudomonas aeruginosa* and *E. faecalis*, in comparison to free chlorhexidine [117]. The antibiofilm activity of chlorhexidine was improved by the nanoparticles.

Another proposition is the use of multi-metallic nanoparticles for dual biofilm penetration and antimicrobial activity. A combination of two or more metallic nanoparticles may be formulated to take advantage of the synergistic activity of the metals [118]. Holden et al. synthesized Ag and gold (Au) bimetallic nanoparticles where the incorporated Au reduced toxicity of Ag towards cells [119]. The Ag/Au nanoparticles could inhibit the growth of *P. gingivalis*. Microscopic images indicated damage of the bacterial membrane induced by the nanoparticles [119]. However, more work is required to provide the synergistic activity against periodontitis and caries causing pathogens, and the potential for reduced cytotoxicity against fibroblasts—possibly through combination of Ag with a benign metal.

The incorporation of Ag nanoparticles into biocide-releasing polymers has also been investigated during the search for dental composites possessing antimicrobial activity. A recent strategy is the incorporation of Ag nanoparticles in 3D printed polymeric dental composite resins which could be further extended to include materials for denture bases, implants and braces [52]. These nanoparticles are thought to exert antimicrobial effects by releasing Ag^+^ ions which coordinate to electron-donating groups, thus disrupting various biochemical processes involving enzymes and DNA [52]. In another study, Cao et al. developed a resin-based dental material with a photocurable core-shell of sliver bromide (AgBr) coupled to a cationic polymer nanocomposite (AgBr/BHPVP) [120]. This composite exerted antibacterial effects against *S. mutans* during the direct contact test through its biocide-releasing mechanism of the Ag^+^ ions as well as a contact-killing mechanism exerted by the cationic polymer and thus showed great promise as an antimicrobial agent which could be incorporated into dental composites, adhesives, cements as well as sealants [120]. The biocompatibility of the AgBr/BHPVP-containing resin disks was assessed using the CKK-8 assay to establish cytotoxicity against RAW 264.7 macrophages. While samples with 0.5 and 1.0 wt% AgBr/BHPVP did not significantly enhance cytotoxicity when compared to pure resin disks alone, 1.5 wt% AgBr/BHPVP reduced cell viability by approximately 50% [120].

More recently, an antibacterial root canal sealer containing dimethylaminohexadecyl methacrylate (DMAHDM), Ag nanoparticles and amorphous calcium phosphate nanoparticles was developed and tested on bacteria-impregnated human dentin blocks [121]. The root canal sealer containing 5% DMAHDM, 0.15% Ag nanoparticles and 30% amorphous calcium phosphate nanoparticles not only reduced the biofilm CFU of *E. faecalis* by approximately 3 logs compared to the control, but also demonstrated acid neutralising capabilities which could prove useful in preventing the growth of anaerobic bacteria. As this root canal sealer contained three bioactive agents it was able to inhibit bacteria through both the release of Ag^+^ from the nanoparticles as well as the contact-killing mechanisms of DMAHDM while simultaneously releasing Ca and P ions which could remineralize root dentin. This sealer thus demonstrated highly favourable antibacterial and remineralisation characteristics that showed great promise in strengthening tooth root structures and promoting favourable outcomes in endodontic therapy [121].

### 5.2. Polymeric Nanoparticles for Drug Delivery and Immunomodulation

Polymeric nanoparticles are most commonly synthesized from chitosan due to its antibacterial properties, ability for pH controllable release, bioadhesion and its biocompatibility and biodegradability [122]. Other polymers used for nanoparticle synthesis include polycaprolactone (PCL) and poly(lactic-co-glycolic acid) (PLGA) as they are inert and do not elicit major cytotoxic effects in mammalian cells [123]. Typically, studies involve the encapsulation of a tetracycline (e.g., doxycycline) and the demonstration of antibacterial activity in vitro against periodontitis causing pathogens and in vivo animal models of periodontitis. Immune modulation is also investigated in instances where the immuno-modulatory antibacterial agent doxycycline is encapsulated. Controlled release as well as stimuli-responsive systems based on these polymers have been synthesized and characterized.

Xu et al. reported the loading of the antimicrobial agent doxycycline into polymeric nanoparticles composed of chitosan and carboxymethyl chitosan [124]. This nanoparticle was anticipated to penetrate the plaque biofilm more effectively and kill the bacteria. The authors reported the bacteriostatic activity of the loaded nanoparticles against *P. gingivalis*. Doxycycline is a well know immuno-modulatory agent, hence this nanoparticle system was also investigated as a host-directed immune-therapy for periodontitis. The nanoparticles were reported to effectively down-regulate the mRNA and protein levels of NLRP3 inflammasome and IL-1β in human gingival fibroblasts [124]. The NLRP3 inflammasome plays an important role in regulating innate immune responses in periodontitis [125]. This study therefore demonstrated the capability of nanoparticles to deliver a drug for both antimicrobial activity as well as immune modulation, towards the treatment of periodontitis. Another tetracycline, i.e., minocycline, has also been loaded into chitosan nanoparticles and a characterization of the anti-inflammatory effects was conducted [126]. The nanoparticles were observed to be taken up into endosomes and finally reaching lysosomes (similar uptake and trafficking pathways for *P. gingivalis*). The nanoparticles were found to induce autophagy which the authors proposed could enhance the therapeutic effectiveness of the nanoparticles through degradation of the intracellular pathogen. The nanoparticles were further shown to exhibit an anti-inflammatory effect, reducing IL-1 mediated activation and downregulation of NF-kB signalling in human gingival fibroblasts [126]. These effects are expected to lessen the pathogen-induced inflammatory burden.

Hu et al. investigated pH activatable nanoparticles composed of N,N,N-trimethyl chitosan coated lecithin liposomes loaded with doxycycline [127]. The nanoparticles were designed to release doxycycline at low pH (typically found in the microenvironment of the plaque biofilm). These nanoparticles showed significantly enhanced bacteriostatic activity against *P. gingivalis* and *P. intermedia*, in comparison to doxycycline alone. These nanoparticles could also disrupt the biofilm and in studies conducted using rats, these nanoparticles regulated the activities of osteoclasts and osteoblasts and eliminated inflammation [127].

Calcium fluoride nanoparticles, synthesized due to their higher deposition of fluoride on the tooth and adsorption onto the biofilm, were further loaded together with lignocaine into mucoadhesive films made from thiolated chitosan for prolonged retention of the nanoparticles in the oral cavity. This system was successfully prepared demonstrating suitable mechanical strength, bioadhesion, drug release and permeation enhancement [101]. Echazú et al. reported the development of pH-responsive biopolymer composites, composed of silica nanoparticles in chitosan hydrogels [128]. The composites were loaded with a plant extract comprising antioxidant properties. The composites were reported to increase fibroblast proliferation thereby providing an environment for bone remineralization. Polymeric nanoparticles of PCL encapsulating the anti-microbial agent triclosan were synthesized by Aminu et al. These nanoparticles were further loaded into a chitosan nanogel containing the non-steroidal anti-inflammatory drug flurbiprofen. An in vivo study in rats demonstrated the dual-activity of the nano-formulation providing resolution of gingival inflammation and reduction of accumulated plaque [129].

To improve drug retention at the biofilm-apatite interface, Sims Jr and co-workers investigated the dual release of myricetin and farnesol encapsulated within tooth-binding nanoparticles of poly(dimethylaminoethyl methacrylate)-b-poly(dimethylaminoethyl methacrylate-co-butyl methacrylate-co-propylacrylic acid) (p(DMAEMA)-b-p(DMAEMA-co-BMA-co-PAA)) [130,131] for antibacterial activity against *S. mutans* biofilms. Improved tooth-binding electrostatic affinity of the system was attributed to bound myricetin on the cationic corona of the nanoparticles, therefore, enabling this delivery system to serve as an in situ drug reservoir at the saliva coated tooth surface for biofilm inhibition in dental caries [131].

An interesting therapeutic modality in the treatment of periodontitis is photodynamic therapy. This approach involves a photosensitizer, which is excited through exposure to light to generate ROS which kill the bacteria. Indocyanine green is such a photosensitizer and Rad et al. reported the loading of indocyanine green into chitosan nanoparticles [132]. Results showed that photodynamic therapy through the nanoparticles led to a reduction in the expression of biofilm formation-related gene (rcpA) of *Aggregatibacter actinomycetemcomitans* and this reduction was far more than that induced by indocyanine alone [132].

It is exciting to note that at least one PLGA nanosphere formulation containing doxycycline has undergone clinical trial testing as an adjunctive therapy in the treatment of chronic periodontitis in individuals with type-2 diabetes mellitus. This study was a parallel, double-blind, randomized, placebo-controlled clinical trial and data for 40 individuals was analysed receiving either a placebo of PLGA nanoparticles or doxycycline loaded nanoparticles. Locally applied doxycycline loaded PLGA nanoparticles favoured cytokine modulation and microbial reduction and were able to additionally reduce pockets and bleeding on probing in the patients [133]. This study shows the clinical promise of polymeric drug loaded nanoparticles as dental materials in the treatment of periodontitis.

A drawback to the inclusion of antimicrobial nanoparticles within dental restorative, treatment and replacement materials is the compromised mechanical integrity attributed to the clustering or leaching-out of the incorporated nanostructures, resulting in the formation of weak regions throughout the material matrix. A study by Makvandi and co-workers investigated the design of a photocurable non-diffusible and non-leaching antimicrobial quaternary ammonium methacrylate resin modified with silica nanoparticles (QMSN) for enhanced mechanical properties [134]. Compared to unmodified commercial dental resin, the QMSN system demonstrated improved flexural strength and modulus with sufficient antimicrobial activity and lower fibroblast cytotoxicity at a concentration of 2.5%. Although higher QMSN concentrations of 5–10% increased antimicrobial activity and mechanical strength, cell viability was greatly reduced to levels in the range of 15–30% [134].

## 6. Antimicrobial and Antifouling Polymers

**Figure 5:**
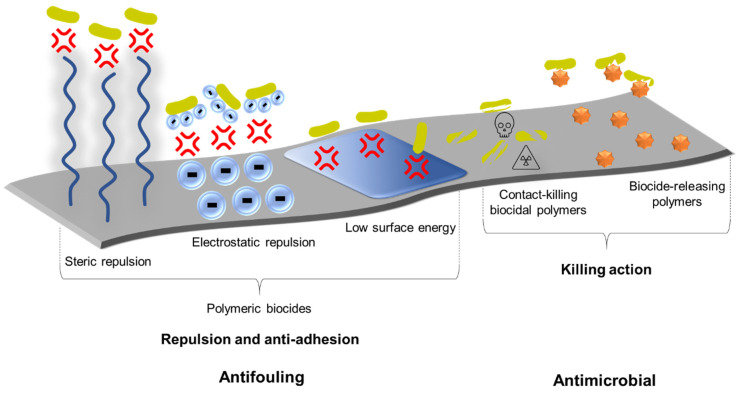
Illustration of the mechanisms of action of the different classes of antimicrobial polymers: polymeric biocides, biocidal polymers and biocide-releasing polymers.

Antimicrobial polymers have gained increasing popularity over the past decade as they offer several advantages over traditionally used antibiotic drugs such as superior efficacy and selectivity, prolonged shelf-life, decreased toxicity, reduced environmental harm and reduced occurrence of antimicrobial resistance as well as being impervious through skin [135,136]. Antimicrobial polymers are classified depending on the mechanism of biocidal activity, as depicted in Figure 5: (1) polymeric biocides, (2) biocidal polymers and (3) biocide-releasing polymers [137,138]. Biocidal polymers possess intrinsic antimicrobial activity within their structure and contain cationic moieties such as quaternary ammonium, tertiary sulfonium, phosphonium and guanidium [138]. The cationic moiety exerts antimicrobial effects by destabilising the negatively charged cell membrane of microbes [139]. Polymeric biocides comprise bioactive repeating units of amino, carboxyl or hydroxyl groups covalently bound to the polymer backbone and act via microbial repulsion or anti-adhesion (rather than killing action) for antifouling properties [138]. Both biocidal polymers and polymeric biocides exert their antimicrobial and antifouling effects, respectively, upon direct contact with the microorganism; whereas biocide-releasing polymers act as a platform for the release of small molecule biocides delivered to the surrounding environment [138,140].

### 6.1. Antimicrobial Quaternary Ammonium Compounds

Of the biocidal polymers, quaternary ammonium compounds (QACs) are heralded as classic and highly effective, yet cytotoxic, disinfectant agents in the medical, pharmaceutical and industrial setting. The mechanism of action of the antimicrobial effects of QACs is a result of contact-killing via electrostatic interaction and subsequent disruption of negatively charged bacterial cell walls by cationic QAC molecules [141]. The first commercial use of QACs for dental applications was initiated by its development in 1994 by Imazato and colleagues with the novel synthesis of the 12-methacryloyloxydodecylpyridinium bromide (MDPB) monomer [10]. The QAC in the MDPB methacrylate-based resin provides disinfectant properties prior to polymerization with bacteriostatic effects post-curing [10]. Previous studies indicated that MDPB displays moderate cytotoxicity in mouse fibroblasts, odontoblast-type cells and human pulpal cells which is considered acceptable for use in dental applications [55,56,57]. Since then, incorporating QACs as monomers and micro- and nanofillers in the synthesis of non-leaching antimicrobial dental composite resins and adhesives for restorative applications have garnered much interest [58].

Recent research in the investigation of QAC monomers for antimicrobial methacrylate dental composite resins and adhesives is focussed on the exploration of novel quaternized materials capable of non-leaching contact-bactericidal effects for maintaining low cytotoxicity and optimal mechanical properties, flexural strength and modulus, after curing. Contact-killing antimicrobial surfaces may be the favourable option as the activity of biocide-releasing polymers is anticipated to be depleted at some point. Biocide-releasing polymers are thus associated with disadvantages including a limited shelf-life and a higher potential to promote antimicrobial resistance as the concentration of the biocide compounds released gradually approaches depletion. Antimicrobial polymers could overcome these problems through the irreversible immobilisation of the biocidal molecules on the polymer [142].

To address compromised mechanical integrity resulting from miscibility of QACs with commercial dental resins, the quaternary ammonium dimethacrylate monomer N,N-bis (2-(3-(methacryloyloxy)propanamido)-ethyl)-N-methylhexadecyl ammonium bromide (IMQ-16) was incorporated into a diurethane dimethacrylate (UDMA)/tricyclodecane dimethanol diacrylate (SR833s) resin system [143]. The IMQ-16-UDAM/SR833s resin demonstrated antimicrobial effects against *S. mutans* yet of comparable mechanical strength to the bisphenylglycidyl dimethacrylate (Bis-GMA)/triethylene glycol dimethacrylate (TEGDMA) resin system in terms of flexural strength and modulus. The authors further hypothesized improved biocompatibility of IMQ-16 loaded polymer systems, compared to Bis-GMA/TEGDMA, owing to lower water solubility [143]. The properties of water solubility and water sorption modulate mechanical strength and biocompatibility of antimicrobial resin systems as water penetration and polymer dissolution results in gradual mechanical failure and the release of potentially cytotoxic unreacted monomers.

The antibacterial mechanism as well as biocompatibility of QACS is further influenced by the alkyl chain length of the compound. It has been known that increasing alkyl chain length, to a certain limit, enhances antibacterial activity whereas any further increases beyond the limit results in reduced activity [139]. In contrast, decreasing chain lengths have been associated with increased biocompatibility. Li and co-workers developed new short-chained quaternized pyridine dimethacrylates derived from niacin (vitamin B3) for the synthesis of an antibacterial dental resin with the objective of enhancing biocompatibility of the system [144]. Although no biocompatibility studies were reported in the paper, the authors anticipate that the presence of niacin would have a positive effect on cell viability and have acknowledged cytocompatibility testing in future [144].

With increased focus being placed on biocompatibility of these much needed antibacterial and antifouling dental restorative materials, Silva et al. investigated keratinocyte cytocompatibility and ultimate tensile strength of myristyltrimethylammonium bromide (MYTAB)incorporated dental resins. MYTAB concentrations of 2% decreased bacterial growth with no adverse effects on tensile strength whereas concentrations of 1% significantly displayed stunted cell viability to a region of 50%. The researchers proposed that a 0.5% concentration would confer sufficient antibacterial activity to the dental resins yet maintain compatible physical and chemical stability and biocompatibility (approximately 90%) [145].

With initial reports of its synthesis, by Li and co-workers, dimethylaminohexadecyl methacrylate (DMAHDM) has stimulated interest in the evaluation of its QAC antimicrobial and biocompatibility properties [146]. An interesting study investigating the *rnc* gene deletion of *S. mutans* with DMAHDM displayed considerable antibacterial effects within a dental resin compared to the activity of the commonly used germicide, chlorhexidine [147]. The reported observations of reduction in biofilm biomass, polysaccharide and lactic acid production, may hold promise for the combined strategy of bacterial gene modification with QAC-incorporation for the fabrication of antimicrobial dental composite resins.

### 6.2. Antifouling Zwitterionic Polymers

As a new generation of polymers, zwitterionic materials have shown promising appeal for the development of novel biocompatible antifouling biomaterials for a range of dental applications involving resins, adhesives, cements, composites, coatings, varnishes and sealants [148,149]. Zwitterionic materials contain both cationic and anionic functional groups yet have an overall neutral charge. Its molecular arrangement mimics the phospholipid bilayer in cell membranes with the hydrophilic heads directed outwards and the hydrophobic tails inwards [149,150,151,152]. As superhydrophilic protein-repellent polymers they contain hydrogen-bond acceptors rather than donors. This enables the material to form a hydration shell via electrostatic interaction which imparts its protein-repellent and antifouling ability [149,153,154]. By inhibiting salivary protein adsorption to dental and mucosal surfaces, bacteria have no base for attachment due to reduced coverage of the salivary pellicle thus resulting in prevention of biofilm formation [151]. The zwitterionic polymer, 2-methacryloyloxyethyl phosphorylcholine (MPC), is an established non-toxic material widely used in biomedical applications as biocompatible coatings for metallic implants, catheters and artificial tissues [154]. Early studies indicated that MPC coatings reduced the adherence of common biomedical device colonizers and periodontal pathogens on plastic coverslips and hydroxyapatite disks, respectively [155,156]. Bioprotective effects of MPC were further evidenced on human oral keratinocytes against inflammation-induced damage from commercial oral disinfectants, such as cetylpyridinium chloride [150]. These studies inspired experimentation of MPC and other zwitterions within dental materials for protein-repelling antifouling properties.

Zhang and co-workers were among the first to investigate MPC for the synthesis of a protein-repellent dental adhesive and composite for the prevention of biofilm-induced secondary caries [13,148]. In addition, this group explored the combination of MPC and DMAHDM, for dual action microbial-repellent and antimicrobial activity, within a glass-based dental resin composite [157]. Combining zwitterionic polymers with QACs may offer enhanced antimicrobial effectiveness of QACs in dental materials. It is postulated that the contact-killing antimicrobial mechanism of QACs may be reduced when dental surfaces are coated with a salivary pellicle [12,146,158]. By inhibiting salivary protein adhesion and reducing salivary pellicle production, contact-killing between the QAC-microbial interface improves while the protein-repellent action of zwitterionic material prevents biofilm formation [148,152,159]. The further inclusion of calcium phosphate into MPC-DMAHDM composites imbues a trio of (1) antimicrobial, (2) antifouling and (3) remineralizing rechargeable properties to dental adhesives, sealers and resins [23,160,161].

With its well-known antifouling and biocompatible appeal as well as its simplicity of incorporation with other materials, several studies emphasized the impact on mechanical properties of dental materials upon MPC integration. Zhang and co-workers reported similar mechanical properties to that of a BisGMA/TEGDMA commercial control at MPC concentrations up to 3% wt whereas concentrations exceeding 4.5% wt led to compromised mechanical integrity [148,157]. Lee et al. showed that MPC incorporated into a commercial surface pre-reacted glass-ionomer filler (SPRG) at concentrations within 1.5–5% wt displayed adequate antifouling and mechanical strength of 80 MPa (conforming to ISO 4049 standards); however, concentrations exceeding 5% wt resulted in significant reduction in flexural strength imparted by increased water solubility, water sorption and wettability [162]. Similarly, an orthodontic bonding agent comprising MPC mixed with mesoporous bioactive glass nanoparticles (MBN) also demonstrated mechanical failure and reduced protein-repellent activity with MPC concentrations exceeding 5% wt due to gelation of the polymer resulting from its affinity to moisture [152]. To avoid mechanical compromise of the bulk dental material, MPC could be photochemically conjugated onto the surface of filled composite resins during the dental procedure, however, this requires dental procedures to be short and simple with the use of safe solvents for rinsing out unreacted monomers in the oral cavity [163].

In addition to MPC, Kwon and colleagues recently investigated another zwitterionic polymer, sulfobetaine methacrylate (SB), in dental resin composites, varnishes and cements to impart antifouling properties and mechanical durability [149,159,162,164]. With 3D-printing as a popular synthesis technique for polymethyl methacrylate (PMMA)-based dental materials, the authors expressed the necessity for evaluations of mechanical integrity while maintaining optimal antifouling action and tissue biocompatibility. In a new study, the addition of MPC or SB in PMMA 3D-printed dental resins demonstrated similar antifouling performance and minimal degradation at zwitterion concentrations in the range of 3–5% wt; although the degradation resulted in reduced mechanical properties, the material strength complied with the International ISO 20795-2 requirements [149].

**Figure 6:**
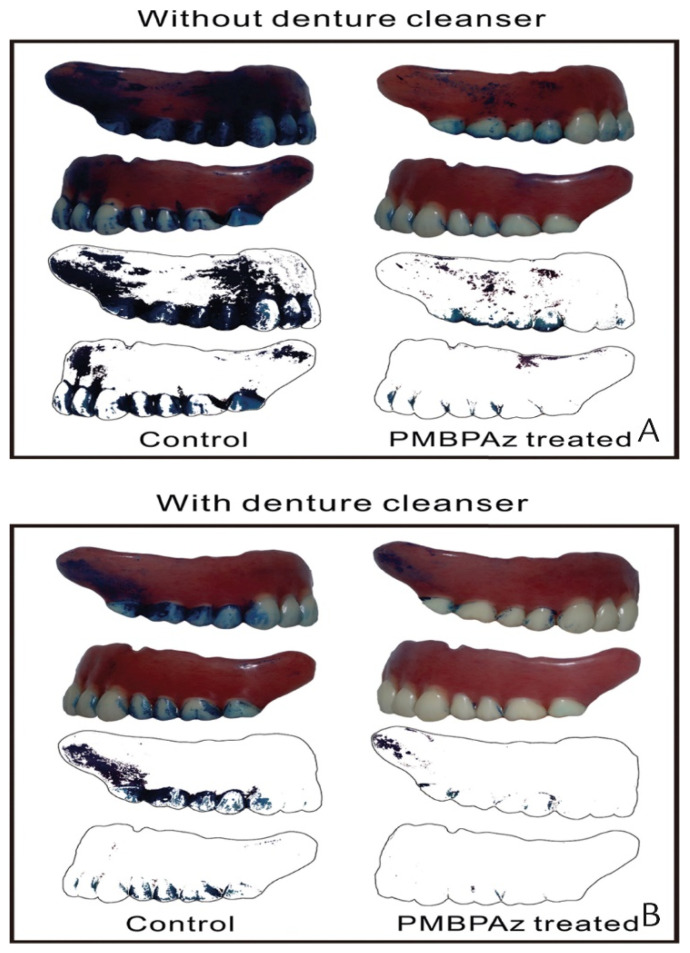
Plaque deposition on maxillary complete denture after 2 weeks. (**A**) Total denture area of polished surface after staining and digitization: left, control; right, PMBPAz treated; and plaque area of polished surface after selection without denture cleanser. (**B**) Total denture area of polished surface after staining and digitization: left, control; right, PMBPAz treated; and plaque area of polished surface after selection with denture cleanser [165]. Reproduced with permission from [165], Elsevier B.V. Ltd. © 2021, RightsLink.

To evaluate its clinical significance, Ikeya et al. polymerized MPC with *n*-butyl methacrylate and photoreactive monomer 2-metahcryloyloxyethyl-4-azidobenzoate (PMBPAz) as an antifouling coating for dentures [165]. The PMBPAz coating showed potential to significantly reduce the percentage plaque index on the coated dentures (as shown in Figure 6) of 11 participants and was able to withstand chemical and mechanical stressors of the oral cavity for 2 weeks—after which time reapplication would be necessary [165]. Demonstrating its clinical performance, a significant reduction in the oral counts of *F. nucleatum* and *Streptococci* spp. was observed within 5 h in a crossover trial consisting of 20 participants after rinsing with 5 mL of a 5% MPC-polymer mouthwash for 20 s [151]. These studies confirming the antifouling effects and safety of MPC in vivo, holds promise for forthcoming studies of this nature, clinical trials and subsequent use of MPC-functionalised dental materials in the clinic.

## 7. Antimicrobial Peptides

Antimicrobial peptides (AMPs) are host-derived endogenous biomolecules forming part of the innate immune system in microorganisms, plants and animals. APMs are short amphipathic cationic peptides featuring broad spectrum antimicrobial activity against Gram-positive and Gram-negative bacteria, fungi and viruses; rapid onset of action, low risk of generating antimicrobial resistance, high biocompatibility and capability of modulating the immune system [32,37,166]. These properties have driven their popularity as promising candidates for the development of antimicrobial surfaces for controlling the growth of cariogenic and periodontic pathogens. Following its uptake via direct penetration or endocytosis, the bactericidal mechanisms of AMPs involve: (1) plasma membrane permeabilization and disruption, (2) intracellular targeting of molecules and processes for inhibition of functional microbial proteins, (3) interruption of DNA and RNA synthesis and (4) immunomodulation and stimulation of non-inflammatory host immune responses for microbial clearance [37,138,167].

### 7.1. Synthetic AMPs for Dental Applications

Naturally occurring AMPs are used as design templates for a wide range of synthetically produced and modified derivatives [138,167,168]. Although natural and synthetic AMPs offer the same bactericidal mechanisms, synthetic derivatives are favoured as they overcome several limitations of natural AMPs such as susceptibility to inactivation by salivary proteolysis, hydrolysis and enzymatic degradation, as well as properties of cytotoxicity, haemolytic activity, poor tissue distribution and improved eradication of multidrug-resistant bacteria [37]. Described below are some examples of recent synthetic AMPs that have shown relevance for biocompatibility, antimicrobial and antifouling action in dental materials. For a comprehensive list of natural and synthetic AMPs over the past several years that have been investigated for dental applications, the reader is directed to a review by Niu et al. [169].

The synthetic peptide, GH12, showed high and rapid in vitro antimicrobial activity against *S. mutans*, *Streptococcus salivarius* and *Streptococcus sobrinus* by inhibiting biofilm formation as well as metabolic activity in biofilms [160,170,171]. In vitro cytotoxicity assays found that GH12 induced low toxicity in human gingival fibroblasts (HGFs) at concentrations of 128 µg/mL over a 2 h incubation period [160]. These properties were confirmed in vivo where GH12 reduced the incidence and severity of caries without causing any damage to the oral mucosa or other signs of ill health in rats [172]. Jiang and co-workers further showed that antimicrobial and antibiofilm activity of GH12 was improved at pH 5.5 than at pH 7.2, therefore, making this AMP a suitable candidate for the development of pH-responsive smart biomaterials in the acidic microenvironment of dental caries [173].

The novel anticancer peptide, ZXR-2, demonstrated antimicrobial activity by rapidly eliminating cariogenic bacteria and inhibiting the formation of *S. mutans* biofilms, however, its effectivity against mature biofilms was limited [174]. Furthermore, at 4 × MIC, ZXR-2 produced 30% lysis of mammalian red blood cells after a 3 h incubation period, thus indicating acceptable cytotoxicity for the prevention and treatment of dental caries [174]. The authors acknowledged the need for improved antibiofilm activity and reduced cytotoxicity of ZXR-2 for its use in dental products such as mouthwashes and toothpastes.

Another synthetic peptide CLP-4 demonstrated stability against salivary proteases, showed strong antibacterial activity against cariogenic *S. mutans,* inhibited biofilm formation and moreover, eradicated established biofilms at 50 µg/mL (at 10 × MIC)—a valuable property that some conventional antibiotics, such as the control erythromycin, do not possess [175]. CLP-4 caused <10% haemolysis in human red blood cells within an hour at concentrations between 8 and 1000 µg/mL and moderate cytotoxicity against human oral fibroblasts at concentration of 5–51 µg/mL after 72 h incubation, hence, its potential or development into an antibiotic for dental caries [175].

To mimic the activity of commonly used fluoride for protection against caries and promotion of its repair, Wang and co-workers, synthesised a novel bifunctional AMP featuring antibacterial remineralising properties [176]. The peptide TVH19 displayed the highest bactericidal activity against *S. mutans* and remineralisation potential among the set of three peptides (TVH19, TDH19 and TNH19) that were synthesised. This AMP was capable of inhibiting biofilm formation at 32 µM, disrupting viability of pre-formed biofilm at 128 µM and maintaining chemical stability of 86.71% after 12 h incubation in human saliva; while in vitro cytotoxicity studies in human oral keratinocytes showed similar cell viability of distilled-deionized water and 512 µM TVH19 [176]. Further studies involving multispecies biofilms, pellicle formation, salivary flow and temperature in a well-simulated oral cavity model is anticipated to precede this first report of bifunctional AMPs [176].

Despite its potent antimicrobial activity and inhibition of biofilms, AMPs perform poorly in the oral cavity due to rapid enzymatic degradation, poor target specificity in solution, anionic protein adsorption and the diluting effects of saliva which render the compound ineffective [167,168,169,170,171,172,173,174,175,176,177,178]. Therefore, strategies are required to improve its physiological in vitro and in vivo stability to harness the maximum antimicrobial and antifouling effects of this exciting class of biomaterials.

### 7.2. AMP Tooth-Binding Strategies

**Figure 7:**
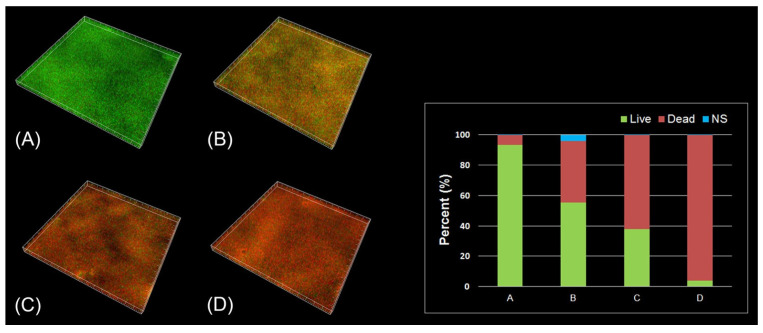
Confocal laser scanning microscope images of *S. mutans* biofilms and the percentage of live/dead cells. (**A**) The biofilms without treatment were performed by the same process as the control; (**B**) The biofilms were cultured for 12 h and then treated with 250 µg/mL hydroxyapatite (HAp)-binding antimicrobial peptide (HBAMP); (**C**) 500 µg/mL HBAMP for 10 min; (**D**) 0.12% chlorhexidine for 10 min. [167]. Reproduced from [167] © Creative Commons CC BY 4.0. Copyright 2016, Springer Nature.

To overcome the challenges of stability in the oral cavity, Huang and co-workers designed a hydroxyapatite (HAp)-binding antimicrobial peptide (HBAMP) conjugate with the AMP KSL-W as a contact-active antibacterial interface on tooth surfaces to inhibit biofilm formation [167]. Rapid and high binding of HBAMP to HAp surfaces provided up to 65% *S. mutans* elimination, as shown in Figure 7, as well as improved stability in saliva [167]. Cytocompatibility was ascertained by proliferation of human gingival fibroblasts, however, exposure of 500 µg/mL HBAMP over 4 h caused disruption in cell membranes and lactic acid dehydrogenase leakage in a time dependant manner [167].

In another study, Zhang et al. grafted the antimicrobial polyphemusin I (PI) to diphosphoserine (DPS) for the synthesis of a tooth-binding DPS-PI AMP. Cytocompatibility studies using bone mesenchymal stem cells (BMSCs) revealed no cytotoxicity after 5 d thus making DPS-PI biocompatible. To determine the ability of DPS-PI to prevent dental plaque biofilm formation in vivo, the incisors of New Zealand white rabbits were coated with DPS-PI for one minute and then washed off with sterile water. Following the administration of a high-sucrose diet for 48 h, DPS-PI was found to significantly reduce the growth of dental plaque biofilms on the rabbit tooth surfaces [179]. Further studies are required to improve the antimicrobial efficacy of DPS-PI and explore its safety profile before its development into oral hygiene dental products for consumers.

A recent study investigated the applicability of a modified dual function (antimicrobial and remineralising activities) AMP, phospherine-grafted-histatin 5, for higher HAp-binding affinity to tooth surfaces in the treatment of decayed teeth [180]. The AMP histatin 5 contains cationic amino acid residues which elicit a bactericidal effect via inducing membrane permeability and damage to intracellular DNA of microbes as well as promote its adsorption to tooth surfaces for forming a bioactive coating with antibiofouling effects and in situ self-healing of carious lesions [180].

### 7.3. AMP Release-Based Strategies

Another approach to protect AMPs in physiological conditions is its incorporation into liquid crystalline systems (LCS) for the controlled release of AMPs as an alternative to antibiotic delivery. This carrier system is useful in the delivery of peptides as it offers the sensitive molecules protection against the harsh degradative mechanisms in the oral cavity [178,181]. Formulating the AMP-LCS with bioadhesive properties further ensures prolonged contact with tooth and oral mucosal surfaces for bacterial and biofilm control as shown by systems loaded with p1025 and KSL-W into a mucoadhesive liquid crystalline carrier for controlled release of the AMPs within the oral cavity via buccal administration [178,181]. Similarly, Aida et al. investigated the AMP β-defensin-3 peptide fragment (D1-23) in an LCS for the prevention of dental caries which demonstrated antibiofilm activity and no cytotoxicity in epithelial cells [182]. In a unique study, KSL-W was incorporated into an antiplaque drug delivery chewing gum with Phase 2 clinical studies showing that the chewing gum successfully inhibited the regrowth of dental plaque in the absence of other oral hygiene methods during a 4-day test period [183].

A gelatinous blend of the AMP NaI-P-113 was injected into the periodontal pocket in patients with periodontitis at a concentration of 20 µg/mL [184]. The release of Nal-P-113 exerted its antimicrobial effects by perforating the cytoplasmic membrane and causing cytoskeletal collapse in *P. gingivalis*, *F. nucleatum*, and *Streptococcus gordonii*. This clinical trial showed that Nal-P-113 administered in gelatine decreased the pocket depth and bleeding index values and thus successfully alleviated gingival inflammation by entering the subgingival dental plaque [184]. It would be interesting to further explore periodontal intrapocket drug delivery systems using AMPs as a replacement for conventional antibiotic drugs due to their reduced propensity of developing microbial resistance. However, being large protein molecules, their permeation through mucosal membranes as well as polymeric matrices and bioactive stability in the oral cavity are key challenges that must be overcome for effective clinical applications.

### 7.4. AMP Immobilisation and Conjugation Strategies

Although AMPs have recently shown great appeal as dental implant coatings and formulation additives in adhesive materials, its successful commercialisation for dentistry remains limited due to toxicity resulting from the need for high effective doses, the potential for immunogenicity and its sensitivity to ion strength [185]. To mitigate these issues, the retention of AMPs on implant and adhesive surfaces via physical adsorption or chemical immobilisation has been proposed to attach AMPs to the biomaterial surface [186].

The antimicrobial peptide, nisin, was incorporated into a commercial adhesive product, Adper Single Bond 2, by means of non-specific adsorption to imbue it with antimicrobial properties. The cured nisin-incorporated dental adhesive successfully demonstrated an inhibitory effect against the growth of *S. mutans* in a dose dependent manner. However, in agar diffusion tests, no significant differences were noted between the cured nisin-incorporated adhesive and the control formulation which may indicate that the antimicrobial effect of nisin is confined within the adhesive system only with no leaching of the peptide [187]. It was previously shown that nisin influences neither the proliferation nor the viability of oral human cells at antimicrobial and anti-biofilm concentrations [188] and did not demonstrate any apparent oral toxicity in F344 rats during a 90-day oral toxicity study [189].

Another study utilised a simple one-step soaking method to adsorb modified AMPs, GH12-M1 and GH12-M2 (containing a single lysine residue), to a commercial dental adhesive formulation blended with ε-polylysine for enhanced antimicrobial activity at the dentin-adhesive interface [190]. Here, ε-polylysine blended in a dental adhesive system facilitated the immobilisation of the AMPs to the surface of the material, however, it would be noteworthy to evaluate the duration of stability, antimicrobial activity and any potential for AMP cleavage resulting in its release in the oral cavity.

Recently, Xie et al. aimed to overcome the limitations of non-specific adsorption techniques, such as reduced antimicrobial efficacy and potential leakage of the peptide out of the resin material, by covalently conjugating the GH12-derived AMP with a monomer commonly used during the formulation of dental adhesives [185]. To further improve the flexibility between the resin material and the peptide, spacer domains were integrated, and the resultant AMPs were then conjugated to methacrylate and finally copolymerised into dental adhesives. The resultant polymethacrylate-based AMP conjugated matrix demonstrated substantial antimicrobial effects against *S. mutans* [185].

Despite being an effective means for non-leaching chemical immobilisation, covalent conjugation may induce structural constraints to AMPs which could adversely alter antimicrobial activity. Another drawback to covalent conjugation procedures is that it can only be conducted before implantation as the processes are usually carried out under harsh conditions that cannot be conducted intraorally [186]. To this end, Moussa et al. exploited the amphipathic properties of AMPs during their attempt to create a peptide-based two-tier protective strategy at the dentin-restoration interface to address the high failure rate of adhesive restorations [191,192]. This hydrophobic system hampers the degradative effects of water and waterborne agents while simultaneously providing antibiofilm protection via coating/priming dentin with GL13K—an amphipathic AMP derived from an oral peptide—to obtain dentin that is resistant to recurrent caries around the bonded restoration areas. In addition, GL13K maintained adequate cell viability of murine embryonic fibroblasts (NIH 3T3) and human dental pulp cells (hDPCs) over a 24 h incubation period at a concentration of 200 µg/mL [191,192].

Another interesting application of AMPs is their incorporation into hydrogel scaffolds for tissue engineering purposes, however, most AMP-containing hydrogels developed to date exhibited low mechanical strength which raises concerns regarding their durability in load-bearing applications in dentistry. Attempts have also been made to integrate AMPs into polymer systems, however, limited success has been reported to date [185]. An alternative application of AMP-containing hydrogel scaffolds is attachment to implant surfaces as bioadhesive coatings. To demonstrate the technique, a hydrogel containing the AMP cateslytin was utilised for antimicrobial efficacy against *P. gingivalis* in the development of peri-implantitis [193]. The supernatant of the cateslytin hydrogel induced no toxicity in HGF-1 human gingival fibroblasts over a 72-h incubation period and did not alter the viability of the fibroblasts. This hydrogel thus demonstrated good cellular biocompatibility and could be attached to the surface of titanium dental implants [193].

Other studies investigating the attachment of AMPs to titanium implant surfaces via covalent binding include GL13K [37]. However, limitations regarding the inadequate control of the orientation and structural conformation of these AMPs have led to the investigation of solid-binding peptide non-covalent immobilisation strategies. These offer the advantage of biomolecular self-assembly of the chimeric peptides onto various implant surfaces and also allows for easier manipulation of the AMP through a variety of biochemical techniques [185,194].

### 7.5. Synthetic Mimics of AMPs as Antimicrobial Polymers

The development of antimicrobial-peptide mimetic polymers is another effective strategy for overcoming the physiological instability of AMP molecules while reducing cytotoxicity towards mammalian cells. The copolymerization of AMPs with conventional polymers (such as polymethacrylates, polyacrylamides, polyamides and polycarbonates) via ring-opening polymerization produces amphiphilic antimicrobial polymers featuring both a cationic and hydrophobic side chain with contact-killing action [195,196]. In the development of AMP-mimetic amphiphilic polymers, it was noted that a high cationic charge and low hydrophobicity of the conventional polymer were critical design parameters for maintaining high bactericidal activity and low cytotoxicity [196]. A study investigating the antimicrobial properties of AMP HHC10 hybrid conjugates with polyphosphoesters, showed the reduction in cytotoxicity of the resulting polymer compared to the free AMP [197]. Therefore, AMP-mimetic polymers may offer the advantages of improved stability and maintenance of AMP antimicrobial activity, low cytotoxicity, ease of processing and manufacture of these rather costly compounds.

Zhou et al. synthesised peptide-mimetic alternating copolymers via copolymerization of ε-*Z*-lysine (cationic hydrophilic moiety) with hexamethylene diisocyanate (hydrophobic moiety). The resulting amphiphilic antibacterial polymer showed no cytotoxicity against mammalian cells and assisted bone repair [198]. This strategy could be evaluated for potential in hard tissue regeneration in periodontitis.

In a study by Takahashi and co-workers, the development of cationic amphiphilic methacrylate polymers via reversible addition-fragmentation chain transfer polymerization yielded polymers with bactericidal and biofilm inhibition activities through eradication of planktonic *S. mutans* bacteria [199]. Compared to chlorhexidine, these amphiphilic polymers displayed superior antimicrobial activity and similar cytotoxicity in human gingival fibroblasts and periodontal ligament stem cells [199].

In other studies, based on the same concept, researchers synthesised methacrylate-ended polypeptides via ring-opening polymerization of *N*-carboxyanhydride (NCA) where UV irradiation with polydopamine (pDA) produced a brush-like polymer coating comprising cationic antimicrobial peptide and antifouling polysarcosine [200]. This AMP-based antimicrobial and antifouling polymer exhibited effective biocidal and antiadhesion activity against several bacterial strains and *C. albicans* over seven days yet maintained adequate biocompatibility in a mouse fibroblast cell line. The resulting dual-function polymer may be immobilised onto various types of biomaterials via the mussel-inspired pDA coating [200]. Another study used a similar approach for the fabrication of nano-structured star-shaped antimicrobial peptide polymers [201]. Although these materials were not developed specifically as dental materials, a similar approach could be investigated for dental applications as antimicrobial and antifouling coatings and nanomaterials.

## 8. Conclusions and Future Considerations

The design of antimicrobial and antifouling materials is highly focussed on the synthesis of direct dental restorative materials such as dental resin composites and adhesives. These constitute the most used materials in dental practice but due to their greater exposure to the microbes of the oral cavity, they are most susceptible to microbial colonization and attack.

Nanomaterials delivered in conjunction or loaded with antibiotics have gained considerable appeal in resins, endodontic sealers, coatings and short-term intra-pocket drug-release systems for the treatment of periodontitis. The cytocompatibility of these systems may be easier to modulate via blending with bio-inert polymeric materials. In some cases, such as with tetracyclines, the released antimicrobial compound has the added benefit of host cell proliferation—a pertinent regenerative feature for the treatment of tissue loss in periodontitis.

Of the polymers with intrinsic antimicrobial and antifouling properties, QACs show promise for sufficient contact-killing action throughout the material bulk whilst maintaining adequate mechanical integrity. Its main challenge of cytotoxicity may be reduced by improving the strength of its covalent bonds to the resin to prevent leaching of monomers. Extensive cytocompatibility studies of leachable monomers in response to mechanical and chemical stresses in dental pulpal, gingival and fibroblastic cells as well as odontoblasts are still required. Within the same class of materials and gaining notable interest in clinical dentistry, are zwitterionic polymeric biocides. Their excellent biocompatibility holds potential for the development of dual function materials when combined with QACs—antimicrobial bactericidal action with antifouling anti-adhesion properties.

As an upcoming generation of new antimicrobial compounds, it is anticipated that AMPs will replace conventional antibiotics and antiseptics, such as chlorhexidine, in dental applications which are predisposed to antimicrobial resistance [184,190,202]. After addressing the key challenges of chemical and physical stability of AMPs in the oral cavity, these compounds may potentially be included in professionally applied treatments and daily homecare dental hygiene products such as toothpastes and mouthwashes.

Antimicrobial and antifouling resin composites, adhesives and coatings intended for long-term wear require extended periods of experimental profiling into the mechanical and chemical stability, biocompatibility and duration of antimicrobial activity. Such studies should be performed in response to prolonged exposure to the physiological and microbiological environment of the oral cavity, the daily activities and oral functions contributing to wear resistance and material life. This will assist in elucidating the performance of these materials in the clinical setting and thus paving the way for its implementation in patients. Properties of compressive strength, moisture sorption and bioerosion should be correlated to cytotoxicity as these factors may contribute to unintentional leaching of unreacted monomers. Experimental designs of future studies should allow for the use of tooth-restoration substrates with constant saliva flow to mimic the oral cavity and its daily wear and tear on these materials [203]. The effects of abrasion on surface properties of dental materials from daily activities of mastication and teeth brushing should also be studied. In addition, biofilm should be cultivated under a high cariogenic potential environment to simulate the use of the materials in caries positive patients. Another factor to consider, is the specific targeting of pathogenic microbial strains to maintain the population of beneficial commensals and a healthy oral microbiome. AMPs may be modified for specific targeting of *S. mutans* in carious lesions through incorporation of a non-specific bactericidal AMP and a targeting moiety consisting of a species-specific, high-affinity binding peptide, such as the *S. mutans* produced pheromone [204]. An alternative strategy investigates the use of narrow-spectrum small molecules (3F1) for specific targeting of *S. mutans* [205].

The emerging trend of multifunctional biomaterials featuring a combination of antimicrobial, antifouling, remineralizing or regenerative properties with microbial specific targeting holds promise for the design of clinically effective dental materials with low-cytotoxic profiles and scalable manufacture for controlling pathogens in the oral cavity and extending the life of dental restorations.

## Figures and Tables

**Table 1:** Essential features of antimicrobial and antifouling materials for dental applications.

Biomaterial Features and Requirements	Importance and Function	References
Biomechanical properties	(i) Surface hardness and toughness to resist indentation and fracture by masticatory forces or abrasive foods. (ii) Compressive, tensile and flexural strength to withstand multidirectional forces of mastication. (iii) Resist long-term wear and tear to minimise maintenance and replacement. (iv) Maintain structural integrity to support tissue regeneration.	[15,16,17]
Physical properties	(i) Thermal stability to resist mechanical deformation and fracture upon temperature induced expansion and contraction. (ii) Minimise water sorption to avoid expansion or swelling and weakened structural integrity via plasticising effects of water.	[18,19]
Biocompatibility and biomimicry	(i) Textural and tribological compatibility with mucous membranes, dentine and dental pulp, bone or enamel. (ii) Minimal systemic and local cytotoxicity against dental tissue, gingiva and mucous membranes from corrosion or leaching of materials. (iii) Bioinertness to avoid foreign body reactions, inflammation or systemic reactions. (iv) Maintain native bone integrity to prevent peri-implantitis and bone resorption. (v) Surface modification to enhance cellular activity, molecular signalling and tissue regeneration. (vi) Optical properties comparative to native teeth for seamless aesthetic appeal.	[13,14,17,20]
Bioerosion and biocorrosion of long-term materials Prevent release of oligomers and monomers; pore formation and layer-by-layer corrosion	(i) Minimise physical degradation by limiting fluid diffusion and absorption into the material leading to formation of a softened network. (ii) Minimise chemical degradation via hydrolysis catalysed by salivary or bacterial enzymes; or acidic and alkaline foods and liquids.	[16,18,19]
Duration of antimicrobial or antifouling activity and rechargeability	(i) Maintenance and efficacy of antimicrobial or antifouling activity in short-term tissue regeneration scaffolds or long-term restorative materials. (ii) Rechargeability of antimicrobial, antifouling or remineralising effects to minimise complete material replacement and maintain long-term efficacy (for example: calcium and phosphate ion-rechargeable dental composites). (iii) Immobilisation strategies for enhanced stability and protection of antimicrobial and antifouling compounds against dilution and inactivation from salivary proteins and enzymes in coating materials.	[21,22,23,24]
Surface properties	(i) High surface energy encourages microbial adhesion. (ii) Rough surface texture encourages cell adhesion of regenerating tissues via increased contact surface area but also encourages microbial adhesion. (iii) Hydrophilic surfaces enhance cellular adhesion of regenerating tissues but prevents microbial adhesion. (iv) Superhydrophobic surfaces prevent both microbial and cellular adhesion of regenerating tissues.	[25,26]
Bioregeneration Encourage guided tissue regeneration Enhance remineralization of hard tissues Control drug and bioactive delivery Tailor biodegradation rates	(i) Roughened or micro-patterned surface topography to assist with cellular adhesion at material-tissue interface. (ii) Delivery of anti-inflammatory compounds, growth factors, stem cells or remineralizing composites to function as therapeutic and chemical cues to stimulate new tissue growth and enhance material-tissue interactions (i.e., osteointegration and osteoinduction). (iii) Biodegradation to match rate of tissue regeneration to avoid occlusion or hindrances in tissue healing.	[14,17]
Fabrication, processing and handling requirements	(i) Scalable fabrication methods for reproducibility and quality. (ii) Ease of material workability upon bio-implantation. (iii) Withstand thermal and chemical sterilization processes and packaging.	[27,28]
